# Mode II Interfacial Fracture Toughness of Multi-Walled Carbon Nanotubes Reinforced Nanocomposite Film on Aluminum Substrate

**DOI:** 10.3390/nano10050904

**Published:** 2020-05-08

**Authors:** Shiuh-Chuan Her, Pao-Chu Chien

**Affiliations:** Dept. Mechanical Engineering, Yuan Ze University, Chung-Li 320, Taiwan; s1005043@mail.yzu.edu.tw

**Keywords:** mode II fracture toughness, multi-walled carbon nanotube, end-notched flexure, strain energy release rate

## Abstract

In this investigation, various loadings of multi-walled carbon nanotubes (MWCNTs) ranging from 0.3–1.0 wt % were incorporated into the epoxy to fabricate the nanocomposites. Nanocomposite film with a thickness of 0.2 mm was deposited on an aluminum substrate through a hot-pressing process. Theoretical expression of the model II strain energy release rate for the film/substrate composite structure was derived. End-notched flexure (ENF) tests were performed to characterize the mode II fracture energy of the composite structure. Experimental results indicate that the elastic modulus, ultimate strength, and mode II fracture energy increase as the MWCNT loading in the nanocomposite increases. In the case of nanocomposite film with 1.0 wt % of MWCNTs, the elastic modulus, ultimate strength, and mode II interfacial fracture toughness are increased by 20.6%, 21.1%, and 54.4%, respectively in comparison with neat epoxy. In addition, the dispersion of MWCNTs in the epoxy-based matrix was investigated using scanning electron microscope (SEM). The SEM images depict that MWCNTs are well dispersed leading to the enhancement of the mechanical properties of the nanocomposite.

## 1. Introduction

Materials play a crucial role in advanced engineering structures. There is always a strong demand to employ novel materials with enhanced properties in industry. Carbon nanotubes (CNTs) have attracted wide attention in the scientific community and industry field owing to their excellent mechanical, electrical, thermal, and chemical properties [1,2,3,4]. Epoxy resins with low cost, dimensional stability, chemical resistance, low weight, and high adhesion have received enormous potential applications in aircraft, aeronautics, and electronic industries. In attempts to enhance the material properties of the epoxy, various types of fillers were incorporated with epoxy resins [5,6,7]. Among nano-fillers, CNTs are considered as ideal filler material to improve the mechanical properties of epoxy due to their outstanding properties such as exceptional elastic modulus, high ultimate strength, large aspect ratio, and superior thermal and electrical conductivities [8,9]. In order to effectively enhance the mechanical properties of epoxy nanocomposites with the addition of CNTs as reinforcement filler, two main challenges have to be solved: (1) poor dispersion or insolubility in organic and inorganic solvents of the individual CNTs because of the van der Waals force, large surface area, and high aspect ratio; (2) poor interfacial adhesion between the epoxy and CNTs reduces the load transfer effectively. To appropriately disperse CNTs in epoxy matrix and strengthen the bonding between CNTs and epoxy matrix, several methods have been proposed, such as chemical functionalization [10,11,12], sonication or shear blending [13,14], and hybridization [15]. Zakaria et al. [16] reported that the flexural modulus, strength, and dielectric constant of multi-walled carbon nanotube (MWCNT)/epoxy composites were increased by 35%, 30%, and 20%, respectively, in comparison with neat epoxy. Esbati and Irani [17] proposed a computational modeling in nanoscale and microscale to investigate the influence of chemical functionalization of CNTs on mechanical properties and failure mechanism of CNT-reinforced nanocomposites.

Based on the increasing demand for many engineering applications, development of flexible and light weight film has been extensively conducted. Nanocomposite films incorporated with CNTs are considered as a good alternate owing to their exceptional mechanical, thermal, and electrical properties. There have been several works which used CNTs as nanocomposite film in the applications of heat transfer and electric interconnection [18,19,20]. Faraji and Aydisheh [21] presented a simple and low cost method to prepare flexible and lightweight PVC-MWCNTs-PANI nanocomposite film as a supercapacitor. Arai et al. [22] fabricated Cu/MWCNT nanocomposite film using pulse reverse electrodeposition method to enhance the thermal conductivity. Kwon et al. [23] prepared a hybrid filler consisting of graphene nanoplatelets (GNPs) and single-walled carbon nanotubes (SWNTs) nanocomposite film as thermoelectric devices which can be used to convert waste heat into electrical energy. Zhang et al. [24] employed carbon nanotube film to protect aircraft composite structures from lightning strike due to the high electrical conductivity of CNTs. Thin films or layers prepared on the substrate can be subjected to different fracture modes, resulting in the reduction of the performance and structural integrity of the corresponding engineering systems. Thin film/substrate composite components can be implemented into a variety of engineering applications such as thermal barrier layers, encapsulation of solar cells, and vessels, among others [25]. To reveal the fracture mode of the film/substrate composite system, a number of studies have been conducted in recent years through experimental, analytical, and numerical methods. Mei et al. [26] presented analytical and finite element methods to study the wrinkling and buckling delaminations of an elastic film on a compliant substrate. Reinoso et al. [27] proposed a 3D nonlinear interface cohesive finite element to study the delamination of a hard film bonded on a soft substrate. Goyal et al. [28] presented a cohesive model to determine the buckling and wrinkling-induced debonding of a thin film deposited on a rigid substrate. CNT-reinforced nanocomposites have great potential to increase energy absorption due to the unique properties of high aspect ratio, large surface area, and superior mechanical properties. Fracture mechanics is a valuable concept to characterize the failure and damage behavior of composite structures. Most of the works related to fracture mechanics are focused on mode I characterization [29,30,31]. Less attention has been devoted to mode II loading, in particular for those of bending or shear loads prevailed. One of the main reasons that may be attributed to the lack of comparable studies on mode II is mainly owing to the difficulty of the experimental tests. Jagannathan et al. [32] investigated the delamination growth of a carbon fiber composite subjected to mode II loading using a three-point bending test on end-notched flexure (ENF) specimens. Quan et al. [33] conducted the end loaded split (ELS) test to investigate the effect of MWCNTs on the model II fracture energy of carbon fiber reinforced epoxy composites. Ayatollahi et al. [34] used asymmetric four-point bend (A4PB) test to study the mode II fracture toughness of the bulk epoxy. de Moura et al. [35] performed the four-point bending test on ENF specimens to evaluate the fracture energy of wood structures subjected to mode II loading.

The demand of high-performance materials in conjunction with continuing device miniaturization has promoted intensive investigation into micro-scale material properties. Mechanical properties of materials in thin film form are significantly different from their bulk counterparts due to the influences of surface and interfacial effects in addition to microstructural changes. Among the various mechanical properties of interest, fracture toughness at the micro-scale is of great importance in the design of devices such as micro-electro-mechanical-systems subjected to mechanical fatigue and wear-resistant thin films. Nanoindentation has been widely used in the last two decades as a high spatial resolution for measuring fracture toughness at small scales. Ast et al. [36] provided an overview of nanoindentation-based techniques in the fracture toughness evaluation at micro-scale utilizing micro-pillar splitting and micro-cantilever bending methods. The test specimens were fabricated by focused ion beam milling (FIB). Sebastiani et al. [37] presented a critical comparison of the techniques by testing a selected group of bulk (single-crystal silicon, Si) and thin film (chromium nitride coating, CrN) materials. They found good agreement of the fracture toughness measured by single micro-cantilever bending and micro-pillar splitting methods on CrN film, while a discrepancy of 25% between the micro-pillar splitting technique and double-micro-cantilever bending. Micro-pillar splitting possesses some advantages over micro-cantilever bending [36,37]: (1) measurement of crack lengths after the test is not required; (2) residual stress in the upper portion of the pillar is totally relaxed when the aspect ratio (height to diameter) is greater than 1.0, which results in a little influence on the toughness measurement; (3) influence of FIB damage is less significant because the FIB damage is surface-localized in a volume that is relatively far from the position of crack nucleation and propagation. Micro-pillar splitting evaluates the fracture toughness from a simple relationship between the critical load at failure (Pc), the pillar radius (R), and the coefficient (γ) related to elastic and plastic properties of the material. Ghidelli et al. [38] investigated the effects of indenter angle varying from 35.3° to 65.3° on the fracture toughness of the TiN and CrN films measured by micro-pillar splitting. They found that the splitting load linearly increases with the increase of the indent angle for a given pillar diameter. However, the changes of the indent angle and pillar diameter did not affect the measured value of fracture toughness. This is a very important result since the use of a sharper indenter reduces the minimum required pillar diameter to have crack initiation, and possibly extends the applicability of the technique to brittle intermetallics and maybe to high-temperature fracture toughness assessment.

In this work, MWCNT/epoxy nanocomposite films were bonded on an aluminum substrate through a hot-pressing process. Tensile tests were conducted to characterize the mechanical properties of the nanocomposite. Theoretical expression of the model II strain energy release rate for the film/substrate composite structure with interfacial edge crack is derived using the theories of fracture mechanics and Euler–Bernoulli beam. Three-point bending tests were carried out on the ENF specimens to evaluate the strain energy release rate of the film/substrate composite structure under mode II loading. The influence of MWCNTs loading on the fracture toughness and tensile properties of the nanocomposite film was investigated through a series of experimental tests.

## 2. Preparation of MWCNT/Epoxy Nanocomposite

The multi-walled carbon nanotubes were received from Uchess Co. (New Taipei City, Taiwan). The diameter and length of MWCNTs varied from 40 nm to 60 nm, and 5 μm to 15 μm, respectively. MWCNTs were prepared via the chemical vapor deposition process with carbon purity >95%. The low viscosity epoxy Mungo 4200A part A and curing agent 4200B part B produced by Uchess Co. (New Taipei City, Taiwan) were used. The weight ratio between the epoxy and curing agent was 2:1 as recommended by the manufacturer. Ultrasonic sonication was a simple and effective method to disperse MWCNTs into epoxy. However, high power or a long period of ultrasonic sonication may induce damage on the surface of MWCNTs. In this work, a series of trial tests were carried out to evaluate the optimal sonication period and power that can uniformly disperse the MWCNTs into epoxy without causing damage on the MWCNT surface. Experimental results indicate that the optimal ultrasonic period and power were 3 h and 120 W, respectively.

**Figure 1:**
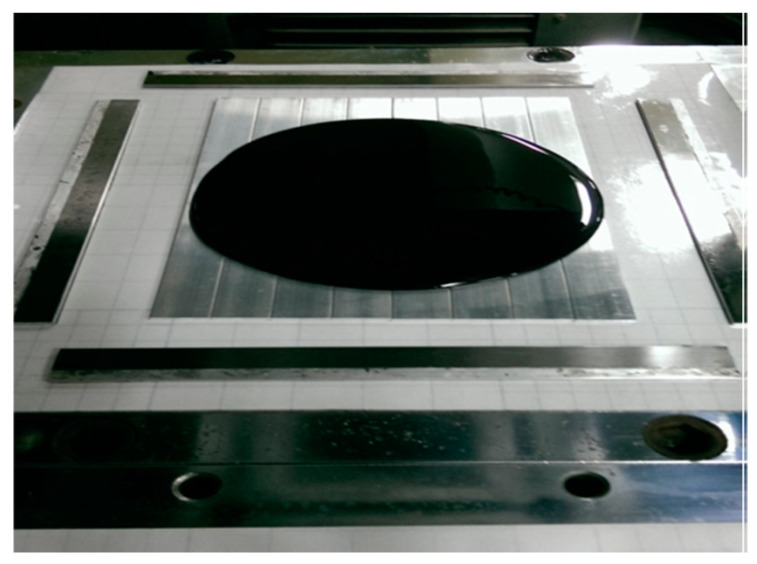
Multi-walled carbon nanotube (MWCNT)/epoxy mixture placed on the aluminum (Al) substrate.

To fabricate the MWCNT/epoxy nanocomposite, MWCNTs were incorporated into liquid epoxy and dispersed through an ultrasonic sonication process for 3 h at a temperature of 40 °C. Then, the curing agent with a weight ratio of 1:2 to the epoxy was added to the mixture, and slowly stirred for 10 min. Consequently, the mixture was degassed in a vacuum chamber at room temperature for 30 min to remove trapped air induced by the stirring. Finally, the MWCNT and epoxy mixture was deposited on an aluminum (Al) substrate, which was held on a steel plate as illustrated in Figure 1. The steel plate was fixed on a hot-pressing machine. The temperature and pressure during the hot-pressing process were 40 °C and 400 N/m^2^, respectively. A spacer was used to control the thickness of the MWCNT/epoxy nanocomposite film on the Al substrate. The nanocomposite was cured in the hot-press mold for 24 h and the film thickness on the Al substrate was 0.2 mm. Various MWCNT loadings of the nanocomposite were prepared with different weight percentages (i.e., 0.3 wt %, 0.5 wt %, 0.8 wt %, and 1.0 wt %) to study the influence of the MWCNT content on the fracture toughness and tensile properties of the nanocomposite.

## 3. Derivation of Mode II Strain Energy Release Rate

**Figure 2:**
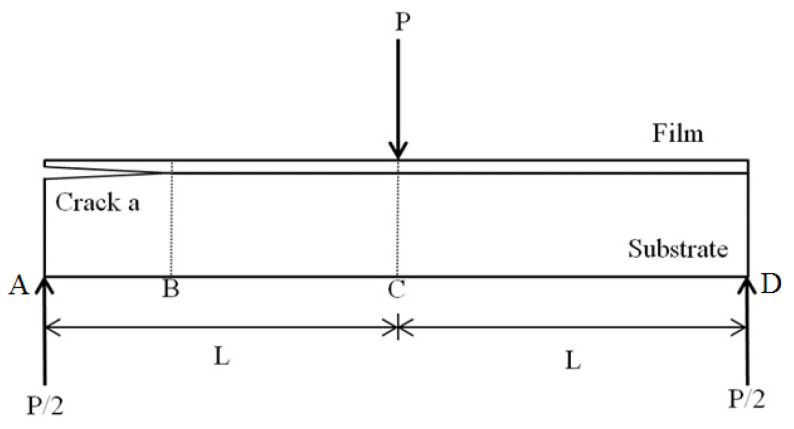
Three-point bending test on end-notched flexure (ENF) specimen.

The mode II fracture analysis of the nanocomposite film/Al substrate composite structure with interfacial edge crack was conducted on an end-notched flexure (ENF) specimen subjected to three-point bending as illustrated in Figure 2. The interfacial crack is located at the edge to adopt the slide deformation due to the flexure of the crack region.

In this work, the strain energy release rate is used to characterize the mode II fracture toughness. The strain energy release rate of the ENF specimen is derived on the basis of the variation of the compliance with the crack propagation. The compliance of the ENF specimen is defined as follows.

(1) $$C=\frac{\delta }{P}$$

where *δ* is the displacement at the loading point, and *P* is the applied load.

**Figure 3:**
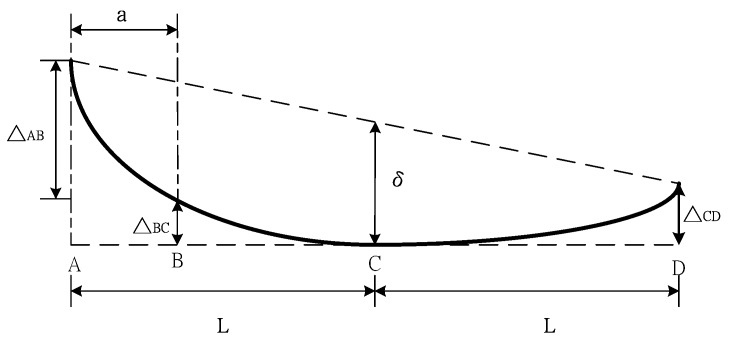
Schematic diagram of the deflection of the ENF specimen subjected to three-point bending.

The deflection of the ENF specimen subjected to three-point bending is schematically illustrated in Figure 3, where *A* and *D* are the two ends of the ENF specimen, *C* is the loading point, and *B* is the interfacial crack tip. The displacement at the loading point *C* can be expressed as

(2) $$\delta =\frac{{∆}_{AB}+{∆}_{BC}+{∆}_{CD}}{2}$$

where ${∆}_{AB},\;{∆}_{BC},\;\mathrm{and}\;{∆}_{CD}$ are the deflections at *A, B,* and *D,* respectively, as shown in Figure 3.

*CA* and *CD* shown in Figure 3 can be modeled as cantilever beams with a fixed end at *C*. Both the cantilever beams are subjected to a load of *P/2* at the free ends *A* and *D*, respectively. The deflections at *B* and *D* can be determined from the Euler beam theory as follows.

(3) $${∆}_{BC}=\frac{\frac{P}{2}{\left(L-a\right)}^{2}\left(3L-L+a\right)}{6\bar{E}\bar{I}}=\frac{P\left(2L^{3}-3aL^{2}+a^{3}\right)}{12\bar{E}\bar{I}}$$

(4) $${∆}_{CD}=\frac{\frac{P}{2}L^{3}}{3\bar{E}\bar{I}}=\frac{PL^{3}}{6\bar{E}\bar{I}}$$

(5) $$\bar{E}\;\bar{I}=\frac{b}{12}\left[E_{f}h_{f}^{3}+E_{s}h_{s}^{3}+3E_{f}E_{s}h_{f}h_{s}\frac{{\left(h_{f}+h_{s}\right)}^{2}}{E_{f}h_{f}+E_{s}h_{s}}\right]$$

where *P* and a are the load and crack length, respectively; *L* is the length of the cantilever beam; $\bar{E}\bar{I}$ denotes the equivalent flexural rigidity of the composite structure containing the nanocomposite film and Al substrate; $E_{f}$ and $h_{f}$ represent the elastic modulus and thickness of the film, respectively; $E_{s}$ and $h_{s}$ are the elastic modulus and thickness of the substrate, respectively.

The deflection $\left({∆}_{AB}\right)$ at *A* consists of two components. One (${∆}_{AB,1})$ is caused by the bending of the beam *AB*, the other $\left({∆}_{AB,2}\right)$ is due to the rotation at point *B*.

(6) $${∆}_{AB}={∆}_{AB,1}+{∆}_{AB,2}$$

The interfacial edge crack separates the film and substrate, leading to double cantilever beams in the delamination region *AB*. Assuming compatible deformation between the double cantilever beams, the loads carried by the film and substrate can be related as follows.

(7) $$\frac{P_{f}}{E_{f}I_{f}}=\frac{P_{s}}{E_{s}I_{s}}$$

where $P_{f}$, $E_{f}$, and $I_{f}$ are the load, elastic modulus, and moment of inertia of the film, respectively; $P_{s}$, $E_{s}$, and $I_{s}$ are the load, elastic modulus, and moment of inertia of the substrate, respectively.

The sum of the loads on the film and substrate is

(8) $$P_{f}+P_{s}=\frac{P}{2}$$

The loads exerted on the film and substrate can be solved from Equations (7) and (8), which yield

(9) $$P_{f}=\frac{E_{f}I_{f}}{2\left(E_{f}I_{f}+E_{s}I_{s}\right)}P$$

(10) $$P_{s}=\frac{E_{s}I_{s}}{2\left(E_{f}I_{f}+E_{s}I_{s}\right)}P$$

The deflection at A due to the bending is readily determined

(11) $${∆}_{AB,1}=\frac{P_{f}a^{3}}{3E_{f}I_{f}}=\frac{Pa^{3}}{6\left(E_{f}I_{f}+E_{s}I_{s}\right)}$$

The slope of the cantilever beam *AC* subjected to a load of *P/2* at the free end *A* may be expressed as follows:

(12) $$\nu^{\prime }=\frac{d\nu }{dx}=\frac{Px}{2\bar{E}\bar{I}}\left(2L-x\right)$$

The slope at *B* can be obtained by substituting *x* = *L* − *a* into Equation (12), which yields

(13) $$\nu^{\prime }=\frac{\frac{P}{2}}{2\bar{E}\bar{I}}\left(L-a\right)\left(2L-L+a\right)=\frac{P}{4\bar{E}\bar{I}}\left(L^{2}-a^{2}\right)$$

The deflection at *A* due to the rotation of the cantilever beam *AC* is the slope at *B* multiplied by the length of the edge crack.

(14) $${∆}_{AB,2}=\nu^{\prime }a=\frac{Pa}{4\bar{E}\bar{I}}\left(L^{2}-a^{2}\right)$$

The deflection at loading point *C* can be determined by substituting Equations (3), (4), (11), and (14) into Equation (2).

(15) $$\delta =\frac{Pa^{3}}{12\left(E_{f}I_{f}+E_{s}I_{s}\right)}+\frac{P\left(2L^{3}-a^{3}\right)}{12\bar{E}\bar{I}}$$

The work exerted on the ENF specimen under three-point bending can be calculated as follows:

(16) $$W=\frac{1}{2}P\delta =\frac{P^{2}}{24}\left(\frac{a^{3}}{E_{f}I_{f}+E_{s}I_{s}}+\frac{2L^{3}-a^{3}}{\bar{E}\bar{I}}\right)$$

The mode II strain energy release rate can be determined as:

(17) $$G_{II}=\underset{\delta A\to 0}{\lim}\left|\frac{\delta W}{\delta A}\right|=\underset{\delta a\to 0}{\lim}\left|\frac{\delta W}{b\delta a}\right|=\frac{P^{2}a^{2}}{8b}\left(\frac{1}{E_{f}I_{f}+E_{s}I_{s}}-\frac{1}{\bar{E}\bar{I}}\right)$$

## 4. Experimental Results

In this work, the tensile properties of the nanocomposite including the elastic modulus, ultimate strength, and fracture strain were evaluated by tensile tests. The mode II fracture energy of the nanocomposite film/Al substrate composite structure with interfacial crack was determined using the three-point bending test. The effect of MWCNT content varied from 0.3 wt % to 1.0 wt % on the tensile properties and fracture toughness of the nanocomposite was examined.

### 4.1. Tensile Properties

**Figure 4:**
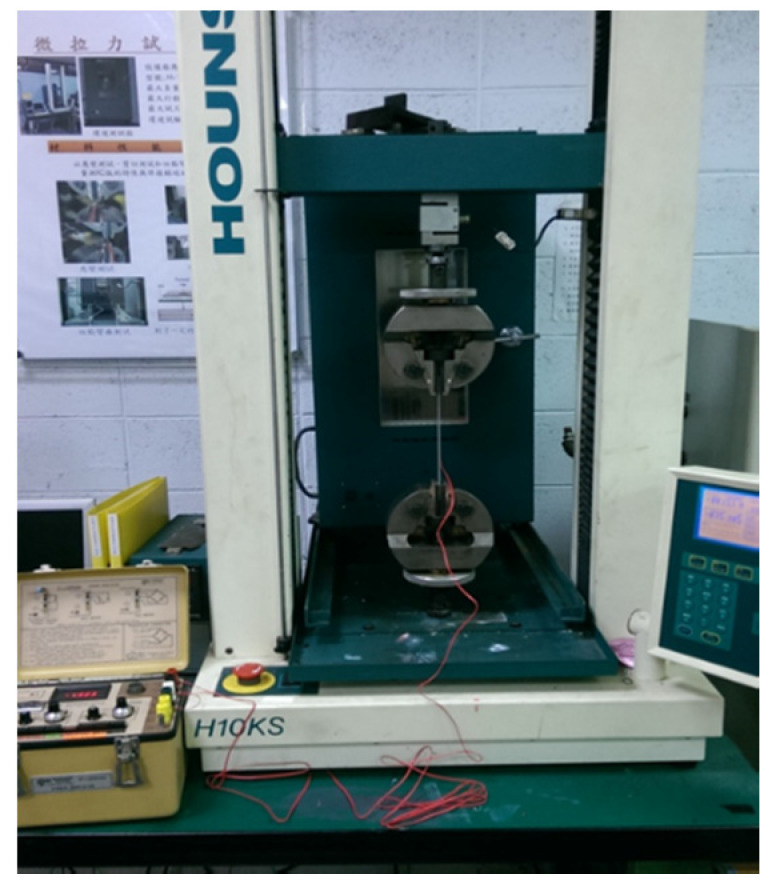
Experimental setup of the tensile test.

**Figure 5:**
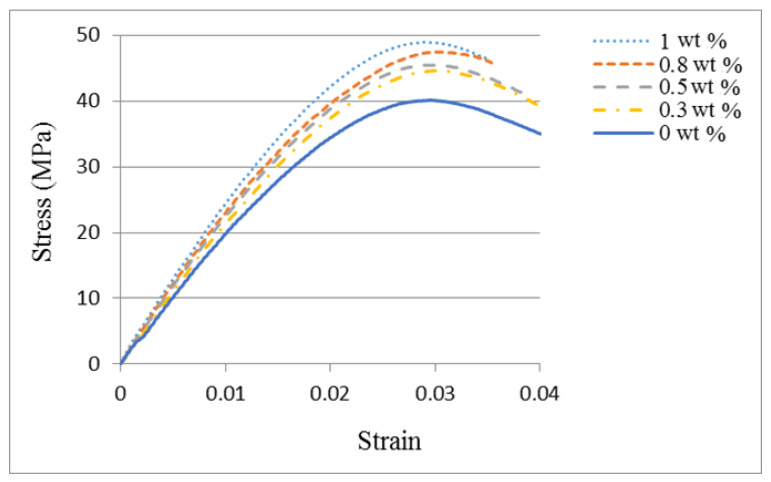
Stress and strain relation for the nanocomposite with different MWCNT loadings.

**Figure 6:**
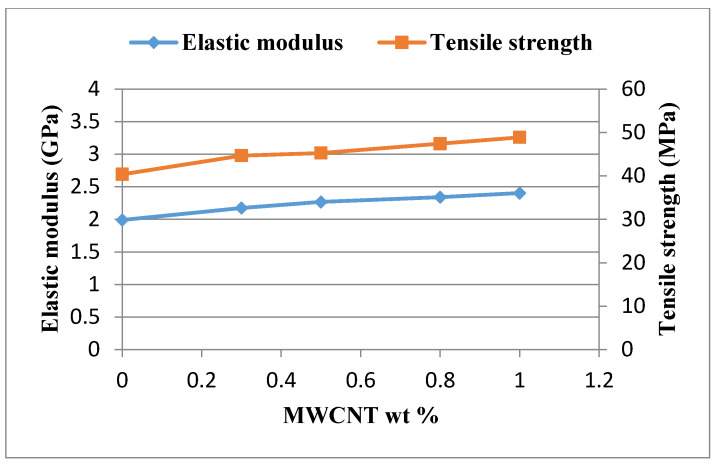
Elastic modulus and tensile strength of the nanocomposite varied with different MWCNT loadings.

**Figure 7:**
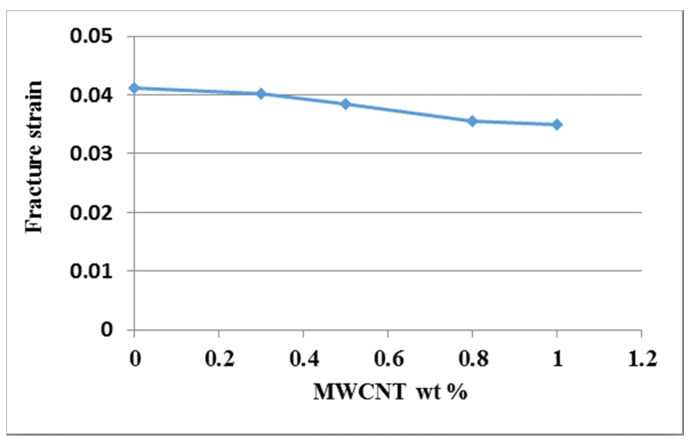
Fracture strain of the nanocomposite varied with different MWCNT loadings.

Tensile tests were conducted in accordance with the ASTM D638 standard to evaluate the mechanical properties of the nanocomposite reinforced with MWCNTs. Figure 4 shows the experimental setup of the tensile test. A Hounsfield 10 KS testing machine of 10 kN capacity was used for the test with the cross bar speed kept at a constant of 5 mm/min. Figure 5 plots the stress and strain relation curves from the tensile tests for the nanocomposites with MWCNT contents varying from 0 wt % to 1.0 wt %. The tensile properties of the nanocomposites such as elastic modulus, ultimate tensile strength, and fracture strain can be obtained from the stress–strain curve. Figure 6 plots the elastic modulus and ultimate tensile strength of the nanocomposite varying with the MWCNT content. It appears that both the elastic modulus and tensile strength of the nanocomposite exhibit a similar trend. Increasing the MWCNT content resulted in an increased elastic modulus and tensile strength. The elastic modulus of the nanocomposite increased from 2.17 GPa to 2.4 GPa as the MWCNT content increased from 0.3 wt % to 1 wt %. The ultimate tensile strength increased from 44.7 GPa to 48.9 GPa while the MWCNT content increased from 0.3 wt % to 1 wt %. The elastic modulus and ultimate tensile strength of the nanocomposite with the addition of 1 wt % MWCNT improved by 20.6% and 21.1% compared with that of neat epoxy, respectively. Figure 7 shows the dependence of the nanocomposite fracture strain on the MWCNT content. An opposite trend was observed in comparison with that of the elastic modulus and ultimate strength. The fracture strain decreased from 0.0402 to 0.0349 as the MWCNT content increased from 0.3 wt % to 1.0 wt %. MWCNT/epoxy nanocomposite with the addition of 1 wt % MWCNT exhibits a reduction of fracture strain by 15.3% compared with neat epoxy. It can be concluded that epoxy reinforced with MWCNTs leads to improvements in the strength and stiffness while the ductility is reduced.

The micro-mechanical properties of the composite constituents are crucial to understand the failure of the composites. Liu et al. [39] demonstrated that the micro-pillar spitting method can be used to determine mechanical properties such as hardness and Young’s modulus of the constituents of aluminosilicate fiber reinforced SiC matrix composites. CNTs have attracted considerable attention for reinforcement material of epoxy due to their low density and excellent mechanical properties (Young’s modulus: 0.5–1 TPa, yield strength: 20–100 GPa). The improvement of mechanical properties can be attributed to the uniform dispersion of the individual CNTs and interfacial bonding with the epoxy matrix. The fracture surfaces from the scanning electron microscope (SEM) images illustrate that MWCNTs uniformly disperse in the epoxy matrix and hold firmly on the epoxy matrix during deformation due to the strong interactions between MWCNTs and epoxy matrix. The mechanical behavior of the nanocomposite induced by the nanoindentation test at the micro-scale is similar with the tensile test at macro-scale [39]. Thus, the trend of mechanical properties of the nanocomposite related to MWCNTs obtained by the tensile test is close to the nanoindentation test.

### 4.2. Mode II Fracture Toughness

**Figure 8:**
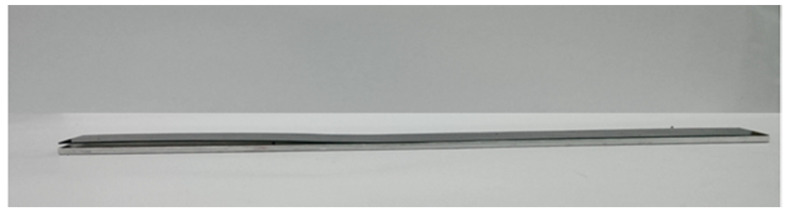
ENF specimen.

**Figure 9:**
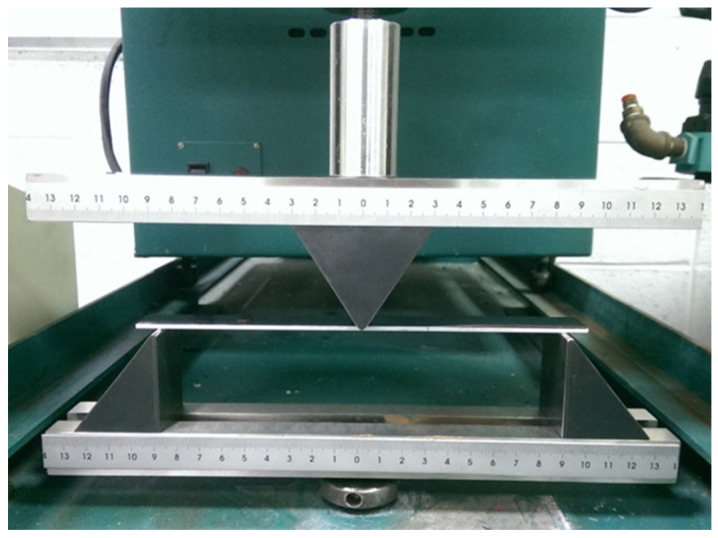
Three-point bending experimental test.

**Figure 10:**
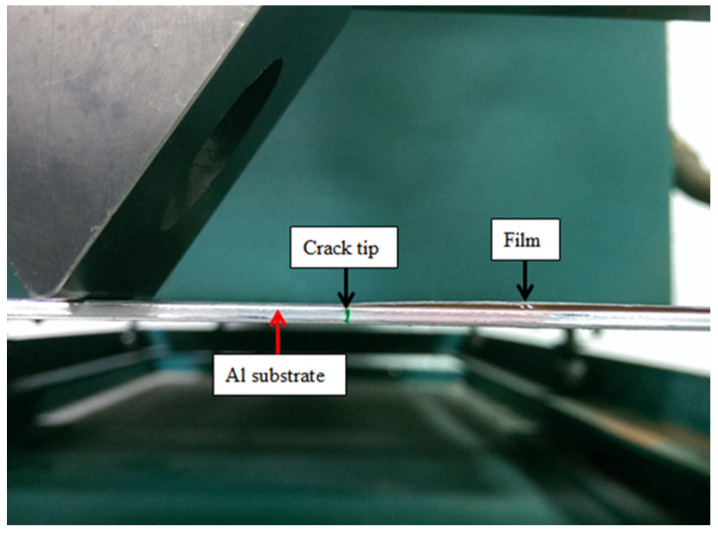
Configuration of nanocomposite film/Al substrate with interfacial edge crack.

**Figure 11:**
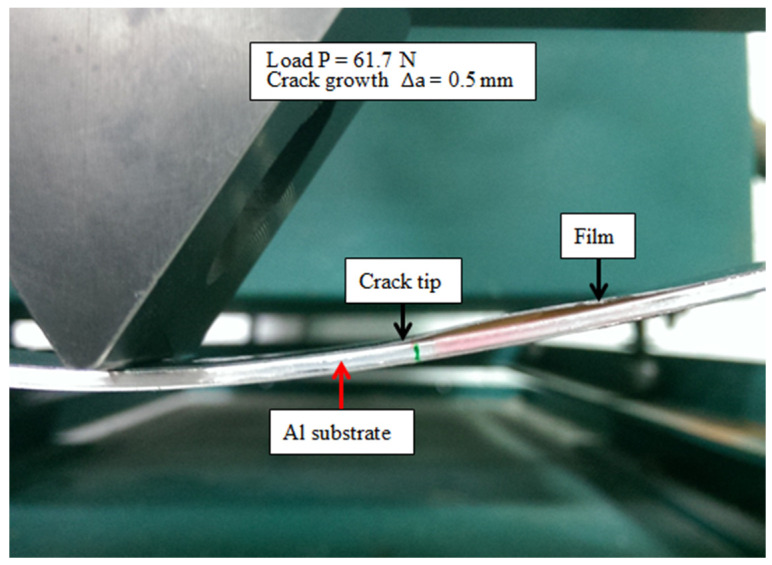
Crack propagation at the critical load.

**Figure 12:**
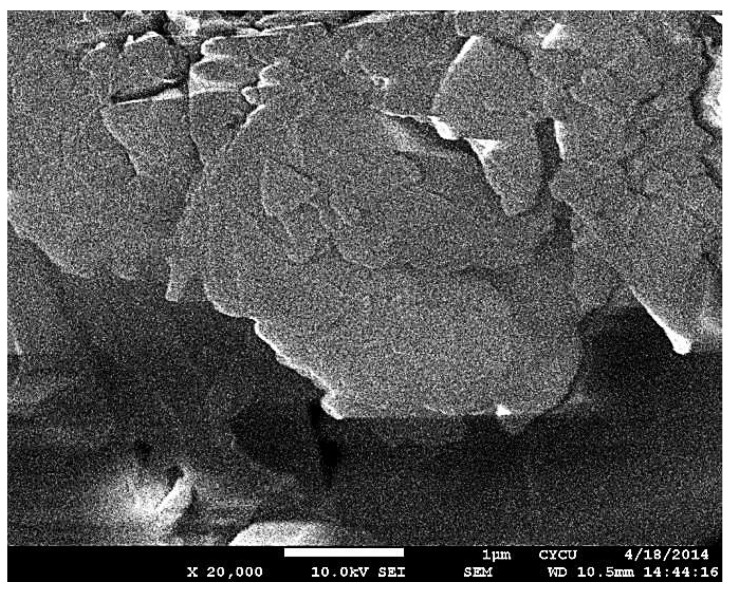
Scanning electron microscope (SEM) image of the neat epoxy.

**Figure 13:**
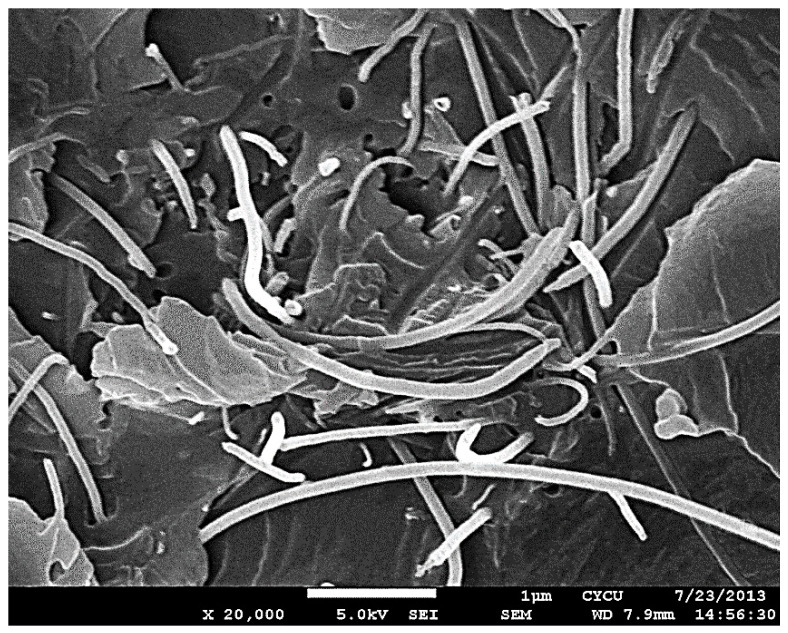
SEM image of the nanocomposite with 0.8 wt % of MWCNT.

The analytical expression of the mode II strain energy release rate for the film/substrate composite structure with interfacial edge crack subjected to three-point bending loading was derived in the previous section. In this section, three-point bending experimental tests were carried out on ENF specimens to estimate the mode II fracture energy of the nanocomposite film on an Al substrate. The dimensions of the Al substrate were 200 × 19 × 2 ${\mathrm{mm}}^{3}$ and the film thickness was 0.2 mm. The length of the interfacial edge crack between the film and substrate was 70 mm. The film/substrate specimen with an interfacial edge crack is shown in Figure 8. The experimental test of the three-point bending is presented in Figure 9. The ENF specimen shown in Figure 10 illustrates the configuration of film/Al substrate with interfacial edge crack. In the experimental test, the load is gradually increased with the cross bar speed 0.5 mm/min to reach the critical load $P_{cr}$ which initiates the crack growth. Red ink was inserted into the interfacial crack region. The crack growth was accompanied by the propagation of the red ink. Thus, the crack initiation can be easily detected. In addition, the experimental test was loaded at a very slow speed 0.5 mm/min and recorded by a video camera. The crack propagation can be visualized through the recorded video which leads to the determination of the critical load. Figure 11 illustrates the crack propagation process. It can be seen that a crack growth of 0.5 mm was observed as the load increased to 61.7 N. The mode II strain energy release rate $G_{IIc}$ is readily calculated by substituting the critical load $P_{cr}$ into Equation (17). Three tests were conducted for each MWCNT content and the results reported in this work are the average of these three tests. Table 1 presents the mode II fracture energy of the nanocomposite film/Al substrate with various loadings of MWCNTs for the nanocomposite film. It appears that the mode II fracture energy is increasing as the MWCNT loading in the nanocomposite film increases. The mode II fracture energy of the nanocomposite film/Al substrate with 1.0 wt % of the MWCNT is significantly improved by 54% in comparison with that of neat epoxy. The improvement of the interfacial fracture toughness may be attributed to the MWCNT reinforcement that enhances the epoxy stiffness as illustrated in Section 4.1. It is considered that several mechanisms affect fracture energy simultaneously such as CNT bridging, CNT pull-out, crack branching, and plastic growth of the polymer matrix. The fracture surfaces of the specimens after tests were examined by SEM. Figure 12 and Figure 13 show the SEM images of the fracture surfaces for the neat epoxy and nanocomposites with 0.8 wt % MWCNT, respectively. It can be observed that the fracture surface of the neat epoxy is relatively smooth compared with the MWCNT-reinforced nanocomposite. This indicates a low resistance to the crack growth for the neat epoxy. Moreover, MWCNTs work as embedded crack arrestors to alter the crack growth, which results in an increase of the interfacial fracture toughness. The investigation of the fracture surfaces from the SEM images as shown in Figure 13 illustrates that failure modes include MWCNT pull-out, MWCNT/matrix debonding, and crack bridging. It can be observed that pulled-out MWCNTs remained on the epoxy matrix after delamination. This observation can be directly associated with the good interactions between MWCNTs and epoxy matrix, which hold them firmly on the epoxy matrix during pull-out of MWCNTs. Thus, MWCNTs on the epoxy surface bridge the crack in nanoscale and improve fracture toughness during crack propagation [40].

It is well-known that the hardness and Young’s modulus of a thin film measured by the nanoindentation technique are heavily dependent on the substrate compliance [41]. The substrate can affect elastic–plastic deformation in the film and thus the crack driving forces. There are interactions between the crack tip and the substrate. To study the influence of the substrate compliance, Sebastiani et al. [37] conducted micro-pillar splitting tests on a film which was deposited on three different substrates: rigid, steel, and polymethyl methacrylate (PMMA), respectively. They reported that the indentation depth increased with the increase of the substrate compliance while the critical load was largely unaffected, with only an 11% difference in the load at failure between the rigid and PMMA substrates. It demonstrated that the fracture toughness measured by the micro-pillar splitting testing is insensitive to the substrate compliance.

## 5. Conclusions

A series of nanocomposite films with various loadings of MWCNTs varying from 0.3 wt % to 1.0 wt % were successfully fabricated and coated on an Al substrate through ultrasonic sonication and hot-pressing processes. Analytical expression of the mode II interfacial fracture toughness for the film/substrate composite structure was derived using the theories of fracture mechanics and the Euler–Bernoulli beam. The effects of MWCNTs on the tensile properties and mode II interfacial fracture toughness were investigated using the tensile test and three-point bending test, respectively. Experimental results demonstrate that the ultimate strength and mode II fracture energy of the nanocomposite with 1.0 wt % of MWCNTs were improved by 21% and 54%, respectively, compared with that of neat epoxy. The improvement of both the ultimate strength and mode II fracture energy is mainly attributed to the increase of the resistance to the crack propagation in the epoxy. MWCNTs act as embedded crack arrestors to alter crack propagation. The increase of the stiffness of the nanocomposite by the addition of MWCNTs also assists in the improvement of fracture energy.

## Figures and Tables

**Table 1:** Mode II strain energy release rate of the nanocomposite film/Al substrate composite structure with different MWCNT loadings.

MWCNT wt %	Specimen 1		Specimen 2		Specimen 3		Average Strain Energy Release Rate $G_{II}$ (J/m^2^)
	Critical Load (N)	Strain Energy Release Rate $G_{II}$ (J/m^2^)	Critical Load (N)	Strain Energy Release Rate $G_{II}$ (J/m^2^)	Critical Load (N)	Strain Energy Release Rate $G_{II}$ (J/m^2^)	
0%	61.7	102.54	62.0	103.54	61.7	102.54	102.87 ± 0.67
0.3%	67.3	121.99	67.3	121.99	67.0	120.91	121.63 ± 0.72
0.5%	70.3	133.11	70.0	131.98	70.3	133.11	132.73 ± 0.75
0.8%	73.7	146.30	73.3	144.71	73.3	144.71	145.24 ± 1.06
1%	77.0	159.69	76.7	158.45	76.7	158.45	158.87 ± 0.82
